# Retinal Optical Coherence Tomography Angiography Parameters Between Patients With Different Causes of Chronic Kidney Disease

**DOI:** 10.3389/fncel.2022.766619

**Published:** 2022-03-11

**Authors:** Meng Hsien Yong, Ming Yean Ong, Kuan Sze Tan, Siti Husna Hussein, Ayesha Mohd Zain, Rozita Mohd, Ruslinda Mustafar, Wan Haslina Wan Abdul Halim

**Affiliations:** ^1^Department of Ophthalmology, Faculty of Medicine, Universiti Kebangsaan Malaysia, Kuala Lumpur, Malaysia; ^2^Department of Medicine, Faculty of Medicine, Universiti Kebangsaan Malaysia, Kuala Lumpur, Malaysia

**Keywords:** optical coherenc tomography angiography, chronic kidney disease, retina, vascular density, perfusion density, macular volume, foveal avascular zone

## Abstract

**Background:**

Chronic kidney disease (CKD) is a major public health issue because of the rising number of patients with the risk of progression to end-stage renal disease. The retinal micro-vasculatures provide a unique window to assess systemic microcirculation. Optical Coherence Tomography Angiography (OCTA) parameters may provide a non-invasive method for systemic correlation. This research aims to compare the association of OCTA parameters in different causes of CKD.

**Methods:**

This is a single-center cross-sectional study on patients with CKD at the Universiti Kebangsaan Malaysia Medical Centre over 2 years. Patients with CKD were divided into three groups: DM group (diabetic CKD), HPT group (hypertensive CKD), and AG group (autoimmune-related glomerulonephritis CKD). The OCTA parameters, namely, the foveal avascular zone (FAZ), vascular density (VD), perfusion density (PD), and macular volume (MV), were measured and recorded using OCTA. Blood and urine analyses were taken as the patient’s CKD profile. The demographic data, the OCTA parameters and the CKD profiles, were analyzed using SPSS version 23.

**Results:**

The right eyes of 232 patients were included. The median age of the control and CKD subjects were 36 and 61 years old respectively. The proportion of the subjects under the control, diabetes mellitus (DM), HPT, and AG group were 30.6, 53.4, 5.6, and 10.4% respectively. There was no significant difference in FAZ, but there is a significant difference in the VD, PD, and MV between the control and CKD groups. There was a statistically significant difference between the three different causes of CKD in VD and PD (*p* < 0.001, *p* = 0.001, respectively). When compared with the control group for VD and PD, there were significant differences between the DM-control group (*p* < 0.001, *p* < 0.001) even when the age variable was considered, but no significant difference when comparing the HPT-control and the AG-control. There was a significant correlation between age, FBS, and HbA1c with VD and PD. There was no significant association between CKD profile and FAZ.

**Conclusion:**

Our study showed the meaningful reduction of VD and PD in patients with diabetes and CKD. However, the use of OCTA to screen or predict CKD in patients living with diabetes mellitus, hypertension, or autoimmune nephritis was not shown to be useful.

## Introduction

Chronic kidney disease (CKD) is a major public health problem because of the rising number of patients with a risk of progression to end-stage renal disease (ESRD), leading to high morbidity and mortality (Levey et al., 2007). CKD is defined as the abnormalities of the kidney structure or function with a glomerular filtration rate (GFR) of less than 60 ml/min/1.73m^2^ present for more than 3 months with health implications (Levin et al., 2013). The leading causes of CKD include diabetes mellitus (DM), hypertension, and glomerulonephritis (Silva et al., 2017; Lerma et al., 2019).

The rate of progression from CKD to ESRD depends on the underlying causes and many other factors. A lower estimated GFR, higher albuminuria, younger age, male sex, lower serum albumin, calcium and bicarbonate, and a higher serum phosphate predict faster progression to kidney failure (Tangri et al., 2011). Besides, proteinuria, elevated systolic blood pressure, heart failure, and anemia are also found to be associated with fast progression (Go et al., 2018).

The retinal micro-vasculatures provide a unique window to assess systemic microcirculation. The pathological and hemodynamic abnormalities in the various systemic causes of CKD may occur throughout the body and post an effect on the retinal vasculature. Retinal vessel changes can be a useful indicator of cumulative and early microvascular damage in systemic micro-vasculopathies (Spaide et al., 2015). Based on the Retinopathy Chronic Renal Insufficiency Cohort (RCRIC) study, 25.3% of patients with CKD showed diabetic, hypertensive, or other retinopathies (Feldman et al., 2003). A similar study found that a lower estimated GFR (eGFR) was associated with higher incidence and severe fundus pathology requiring immediate referral (Grunwald et al., 2010). A study showed that retinal arteriolar narrowing is associated with CKD, independent of diabetes and hypertension (Sabanayagam et al., 2009). However, a study done by Amy et al. concluded that hypertension has significantly narrower retinal arterioles, but no significant associations between retinal vascular parameters and CKD (McGowan et al., 2015).

Further studies are needed to compare the association of the retinal microvasculature abnormalities in CKD and whether there are differences among the various causes of CKD. Traditional fluorescein angiography (FA) and indocyanine green angiography (ICGA) are used to investigate the status of the retinal and choroidal vascular systems. However, FA and ICGA have limitations as they need intravenous dye administration, are time-consuming and are unable to provide 3-dimensional (3-D) images. A study reported there are potentially serious side effects following intravenous sodium fluorescein (Lopez-Saez et al., 1998). The introduction of OCTA is a new non-invasive method for detecting and quantifying retinal microcirculation without the use of dye but motion contrast.

## Methodology

### Study Design

This single-center, cross-sectional study was conducted in the Universiti Kebangsaan Malaysia Medica Centre (UKMMC) between October 2019 and May 2021. Ethical approval was obtained from the UKM Medical Research and Ethics Committee under research code: GGPM-2019-023 before data collection. This research aimed to compare the association of OCTA parameters in different types of CKD. Moreover, this study aimed to investigate the association of OCTA parameters with CKD profile in different types of CKD.

### Inclusion and Selection of Patients

Patients with ages ranging from more than 18 years old and less than 75 years old with CKD who are on regular follow-up at UKMMC were enrolled in this study. The exclusion criteria were dialysis or ESRD patients, patients with renal transplant, patients with underlying malignancy, patients who are in active infection, pregnancy, inadequate quality of OCTA image (signal strength < 6), and patients who are not able to give consent. All subjects have no pre-existing eye diseases and are not on the regular care in the eye clinic. Subjects with underlying DM have no pre-existing diabetic retinopathy or maculopathy based on their latest yearly fundus photo screening done by the primary care physician.

The patients with CKD were divided into three groups: DM group (diabetic CKD), HPT group (hypertensive CKD), AG group (autoimmune-related glomerulonephritis CKD).

Chronic kidney disease cases were defined based on the Kidney Disease: Improving Global Outcomes (KDIGO) guideline as eGFR of <60 ml/min/1.73 m^2^ that is present more than three months with or without evidence of kidney damage (Levin et al., 2013). The severity of CKD was calculated from serum creatinine by using the CKD Epidemiology Collaboration equation (Levey et al., 2009). Patients with diabetes and CKD are defined as patients with DM and no other significant comorbidities that could interfere with the retinal microvasculature.

The CKD subjects were categorized under the DM group (diabetic CKD) when he or she was previously diagnosed DM patients or had his/her venous fasting plasma glucose ≥7.0 mmol/L or 2-h post-prandial glucose ≥11.1 mmol/L if symptomatic, or a positive repeated glucose value in asymptomatic patients (Malaysian Endocrine & Metabolic Society [MEMS], 2015).

Hypertensive CKD patients are defined as patients with hypertension with no other significant systemic diseases that would become a confounding factor. The CKD subjects were categorized under the HPT group (hypertensive CKD) when the CKD subjects were previously diagnosed for hypertension or having a persistent elevation of systolic blood pressure of 140 mmHg or greater and/or a diastolic pressure of 90 mmHg or greater, taken twice on two separate occasions (Malaysian Society of Hypertension [MSH], 2018).

The CKD subjects were categorized under the AG group (autoimmune-related glomerulonephritis CKD) when he or she was previously diagnosed as having systemic lupus erythematosus (SLE) with kidney involvement or IgA nephropathy. Patients with SLE and renal involvement were recruited when he or she were previously diagnosed with SLE by a rheumatologist and/or based on European League Against Rheumatism (EULAR)/American College of Rheumatology (ACR) criteria (Aringer, 2019). Patients with IgA nephropathy were recruited when he or she was previously diagnosed as having IgA nephropathy by a nephrologist.

The control group consisted of healthy subjects with no visual symptoms, absence of retinal disease, no major comorbidities such as DM, hypertension, hypercholesterolemia, and autoimmune diseases.

All patients who fulfill inclusion criteria will be informed about the study protocol and written informed consent was obtained from each patient who participated in this study.

### Sample Size

The sample size was calculated based on G power version 3.1.9.7. It was based on the effect size of 0.25, the power of a statistical test of 0.8, and statistical significance at alpha 0.05 with four groups; the calculated sample size was 180 (45 participants per group).

### Examination Performed

The subjects were interviewed for demographic data (age, gender, race), past medical history, treatment history, and current therapy.

**FIGURE 1:**
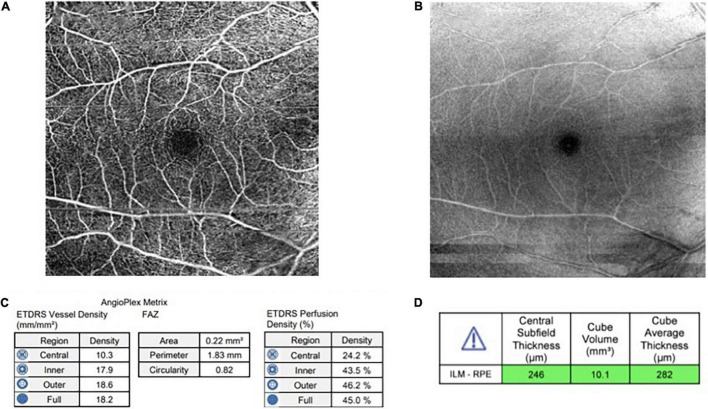
**(A)** An optical coherence tomography angiography (OCTA) image of superficial layer (6 mm × 6 mm) **(B)** An OCTA image of structure at superficial layer **(C)** Angioplex Metrix toolbox which provides automated value for foveal avascular zone (FAZ) (mm^2^), vessel density (mm/mm^2^), and perfusion density (%) **(D)** Macular volume from automated cube volume (mm^3^).

The investigations included CKD profile and OCTA. The most recent three months’ laboratory data was collected while the patients without a most recent three months’ laboratory data proceeded with peripheral blood collection for laboratory analysis on the day of examination. Peripheral blood (15–20 ml) was collected from patients in a sterile container (EDTA, Plain, and Na fluoride/K oxalate tube) and sent for CKD profile. The CKD profile consists of a few tests, namely, full blood count (FBC), fasting blood sugar (FBS), glycosylated hemoglobin (HbA1c), high-density lipoprotein (HDL), low-density lipoprotein (LDL), total cholesterol, triglyceride, serum urea, serum creatinine, serum alanine transferase (ALT), serum alkaline phosphatase (ALP), serum albumin, serum calcium, serum phosphate, sodium, potassium, and urine protein creatinine index (UPCI). The OCTA was conducted on both eyes by using Cirrus HD-OCT 500 (Carl Zeiss, Germany) after dilating the pupils with 1% tropicamide and 2.5% phenylephrine hydrochloride. The OCTA scans (6 mm × 6 mm) centered on the fovea were obtained. Cirrus OCT Angiography provided images of the retinal and choroidal vasculature. The results were interpreted by two ophthalmologists in UKMMC. OCTA microvascular parameters measured include foveal avascular zone (FAZ), parafoveal vascular density (VD), parafoveal perfusion density (PD), and macular volume (MV) (Figure 1). The right eye was chosen for the final analysis, the left eye was only chosen if the scan of the right eye is suboptimal or invalid.

### Statistical Analysis

Kruskal-Wallis test was performed to determine the relationship between causes of CKD and OCTA microvascular parameters whereby the OCTA parameters were not normally distributed. The OCTA parameters between different causes of CKD and control were compared using the Mann-Whitney test. The association between OCTA parameters and CKD profile was determined by using Spearman’s correlation. Quade’s ANCOVA was used to control the covariate in the analysis of OCTA parameters between groups. The normality of the variables was tested with the Kolmogorov-Smirnov test. A two-tailed *p*-value < 0.05 was considered statistically significant. All the statistical analyses were performed using the IBM SPSS Statistics version 23.

## Results

### Demographic Data

There were 232 subjects enrolled in this study. The study subjects consisted of 71 (30.6%) control subjects and 161 (69.4%) CKD subjects. Among the CKD subjects, it consisted of 124 (53.4%) diabetic CKD subjects, 13 (5.6%) hypertensive CKD subjects, and 24 (10.4%) autoimmune-related glomerulonephritis CKD. The median age of control subjects and CKD subjects were 36 and 61 years old respectively. The study population is made up of 109 (47.0%) male subjects and 123 (53%) female subjects. The study subjects consisted of 149 (64.2%) Malay, 54 (23.3%) Chinese, 28 (12.1%) Indian, and 1 (0.4%) other. Tables 1, 2 show demographic data.

**TABLE 1:** Demographic data, optical coherence tomography angiography (OCTA) parameters, and chronic kidney disease (CKD) profiles in chronic kidney disease group and control group.

Variables	Control (*n* = 71)	CKD (*n* = 161)	*p*-Value
Age (median)	36	61	<0.001
Gender, male:female, *n* (%)	19:52 (26.76:73.24)	90:71 (55.90:44.10)	<0.001
Race			0.073
Chinese, *n* (%)	16 (22.5)	38 (23.6)	
Indian, *n* (%)	3 (4.2)	25 (15.5)	
Malays, *n* (%)	52 (73.2)	97 (60.2)	
Others, *n* (%)	0 (0.0)	1 (0.6)	
**OCTA parameters**			
FAZ size, mm^2^			0.228
Mean ± SD	0.277 ± 0.112	0.255 ± 0.113	
Median	0.260	0.240	
Range	0.070–0.051	0.040–0.540	
VD, %			<0.001
Mean ± SD	17.98 ± 1.25	16.16 ± 2.91	
Median	18.30	17.10	
Range	14.30–19.50	6.00–21.90	
PD, %			<0.001
Mean ± SD	44.20 ± 3.11	39.72 ± 7.35	
Median	45.30	42.20	
Range	34.70–47.70	13.10–48.70	
MV, mm^3^			<0.001
Mean ± SD	10.23 ± 0.54	9.76 ± 0.62	
Median	10.30	9.80	
Range	9.20–11.40	7.80–11.10	
**CKD profile**			
Urea, mmol/L			<0.001
Mean ± SD	4.25 ± 1.05	9.76 ± 4.86	
Median	4.40	8.20	
Creatinine, μmol/L			<0.001
Mean ± SD	68.83 ± 11.10	196.47 ± 97.53	
Median	64.65	169.90	
UPCI, g/mmol			<0.001
Mean ± SD	0.01 ± 0.01	0.18 ± 0.27	
Median	0.01	0.08	
FBS, mmol/L			<0.001
Mean ± SD	5.17 ± 0.87	7.13 ± 3.43	
Median	4.94	6.01	
HbA1c, %			<0.001
Mean ± SD	5.67 ± 0.49	7.42 ± 1.93	
Median	5.50	7.20	

*FAZ, foveal avascular zone; VD, parafoveal vascular density; PD, parafoveal perfusion density; MV, macular volume; SD, standard deviation; CKD, chronic kidney disease; DM, diabetic CKD; HPT, hypertensive CKD; AG, autoimmune-related glomerulonephritis CKD; UPCI, urine protein creatinine index; HbA1c, glycosylated hemoglobin; FBS, fasting blood sugar; OCTA, optical coherence tomography angiography.*

**TABLE 2:** Demographic data, optical coherence tomography angiography (OCTA) parameters, and chronic kidney disease (CKD) profiles in diabetes mellitus (DM) group, HPT group, AG group, and control group.

Variables	Control (*n* = 71)	DM (*n* = 124)	HPT (*n* = 13)	AG (*n* = 24)	*p*-value
Age (median)	36	61.5	58	49.5	<0.001
Gender, male:female, *n* (%)	19:52 (26.76:73.24)	74:50 (59.68:40.32)	9:4 (69.23:30.77)	17:7 (70.83:29.17)	<0.001
Race					0.104
Chinese, *n* (%)	16 (22.54)	31 (25.00)	1 (7.69)	6 (25.00)	
Indian, *n* (%)	3 (4.23)	23 (18.55)	0 (0.00)	2 (8.33)	
Malays, *n* (%)	52 (73.24)	69 (55.65)	12 (92.31)	16 (66.67)	
Others, *n* (%)	0 (0.00)	1 (0.81)	0 (0.00)	0 (0.00)	
**OCTA parameters**					
FAZ size, mm^2^					0.114
Mean ± SD	0.277 ± 0.112	0.249 ± 0.110	0.319 ± 0.109	0.250 ± 0.130	
Median	0.260	0.240	0.320	0.230	
Range	0.070–0.051	0.040–0.520	0.140–0.520	0.040–0.540	
VD, %					<0.001
Mean ± SD	17.98 ± 1.25	15.66 ± 3.00	18.15 ± 0.86	17.65 ± 2.08	
Median	18.30	16.70	18.30	17.80	
range	14.30–19.50	6.00–19.50	17.10–19.40	12.90–21.90	
PD, %					<0.001
Mean ± SD	44.20 ± 3.11	38.63 ± 7.77	44.69 ± 1.99	42.61 ± 4.69	
Median	45.30	41.25	45.40	42.95	
Range	34.70–47.70	13.10–48.20	42.30–47.40	32.00–48.70	
MV, mm^3^					<0.001
Mean ± SD	10.23 ± 0.54	9.76 ± 0.62	9.89 ± 0.41	9.70 ± 0.70	
Median	10.30	9.80	9.80	9.90	
Range	9.20–11.40	8.00–11.10	9.10–10.50	7.80–10.90	
CKD profile					
Urea, mmol/L					<0.001
Mean ± SD	4.25 ± 1.05	9.55 ± 5.09	10.23 ± 3.87	10.20 ± 4.71	
Median	4.40	7.80	9.80	9.40	
Creatinine, μmol/L					<0.001
Mean ± SD	68.83 ± 11.10	190.00 ± 103.65	233.58 ± 84.86	194.27 ± 79.70	
Median	64.65	152.00	199.35	180.70	
UPCI, g/mmol					<0.001
Mean ± SD	0.01 ± 0.01	0.21 ± 0.31	0.08 ± 0.06	0.10 ± 0.10	
Median	0.01	0.09	0.07	0.07	
FBS, mmol/L					<0.001
Mean ± SD	5.17 ± 0.87	7.90 ± 3.62	5.32 ± 0.52	5.51 ± 2.71	
Median	4.94	6.86	5.40	4.95	
HbA1c, %					<0.001
Mean ± SD	5.67 ± 0.49	8.13 ± 1.89	5.81 ± 0.35	5.88 ± 0.83	
Median	5.50	7.70	5.75	5.70	

*FAZ, foveal avascular zone; VD, parafoveal vascular density; PD, parafoveal perfusion density; MV, macular volume; SD, standard deviation; CKD, chronic kidney disease; DM, diabetic CKD; HPT, hypertensive CKD; AG, autoimmune-related glomerulonephritis CKD; UPCI, urine protein creatinine index; HbA1c, glycosylated hemoglobin; FBS, fasting blood sugar; OCTA, optical coherence tomography angiography.*

### Optical Coherence Tomography Angiography Findings

There was no significant difference in FAZ between the control group and the CKD group, the median of FAZ of the study population was.245mm^2^. There was a significant difference in VD, PD, and MV between the control group and the CKD group. The median of VD, PD, and MV for the control group were 18.3, 45.3, 10.3 mm^3^, respectively. While the median of VD, PD, MV for the CKD group were 17.1, 42.2, 9.8 mm^3^, respectively (Table 1).

Based on the Kruskal-Wallis test (Table 3), there was a statistically significant difference in between the 3 different causes of CKD in VD and PD (*p* < 0.001, *p* = 0.001, respectively). By comparing each of the different causes of CKD to the control group using the Mann-Whitney test, we found that there was no significant difference between the different causes of CKD and FAZ after age adjustment. However, there was a significant difference between different causes of CKD and MV (DM-control: *p* < 0.001, HPT-control: *p* = 0.042, AG-control: *p* = 0.003). On the other hand, there were significant differences between the DM group and the control group in terms of VD and PD (*p* < 0.001, *p* < 0.001). Quade’s ANCOVA was performed whereby the age was considered and VD and PD were still significantly associated with the DM group (*p* < 0.001, *p* < 0.001). However, there was no significant difference in terms of VD and PD when comparing HPT group with the control group and the AG group with the control group. On the other hand, there was a significant and strong correlation between age and VD and PD (*p* < 0.001, *r*: −0.349; *p* < 0.001, *r*: −0.329), while there was a weak correlation between age and MV (*p* = 0.004, *r*: −0.186).

**TABLE 3:** The *p*-value of chronic kidney disease (CKD) group, diabetes mellitus (DM) group, HPT group, AG group vs. control, and CKD group to optical coherence tomography angiography (OCTA) parameters.

Variables *N* = 232	DM vs. control (*p*-value)	HPT vs. control (*p*-value)	AG vs. control (*p*-value)	CKD vs. control (*p*-value)	CKD (*p*-value)*
FAZ	0.131	0.192	0.389	0.228	0.106
VD	<0.001	0.985	0.625	<0.001	<0.001
PD	<0.001	0.887	0.286	<0.001	0.001
MV	<0.001	0.042	0.003	<0.001	0.773

*p-value of CKD group to OCTA parameters based on Kruskal-Wallis test.

### Chronic Kidney Disease Profiles

Spearman correlation was used to study the association between OCTA parameters and CKD profile. FBS was significantly associated and fairly correlated with VD and PD (*p* < 0.001, *r*: −0.350, *p* < 0001, *r*: −0.313 respectively). Moreover, HbA1c was also significantly associated and fairly correlated with VD and PD (*p* < 0.001, *r:* −0.483, *p* < 0.001, *r*: −0.450 respectively). There was no significant association between CKD profile and FAZ. Table 4 shows chronic kidney disease profiles.

**TABLE 4:** The *p*-value and Spearman’s correlation coefficient of chronic kidney disease (CKD) profile to optical coherence tomography angiography (OCTA) parameters.

Variables *N* = 232	FAZ	VD	PD	MV
FBS	0.271	<0.001 (*r*: −0.350)	<0.001 (*r*: −0.313)	0.013 (*r*: −0.167)
HbA1c	0.077	<0.001 (*r*: −0.483)	<0.001 (*r*: −0.450)	0.001 (*r*: −0.223)
UREA	0.085	0.012 (*r*: −0.166)	0.017 (*r*: −0.157)	<0.001 (*r*: −0.233)
CREATININE	0.296	0.010 (*r*: −0.169)	0.013 (*r*: −0.163)	<0.001 (*r*: −0.265)
UPCI	0.599	0.094	0.115	0.115

*r, Spearman’s correlation coefficient.*

## Discussion

Firstly, there is no significant difference in FAZ between the control and the patients with CKD in this study, and there is no difference between different causes of CKD. This observation is further supported by the absence of correlation between FAZ with the level of UPCI, urea, and creatinine (significantly higher in CKD group), FBS and HbA1c (considerably higher in DM group), and even age (significantly lower in the control group). This finding was supported by previous studies done by Oliverio et al., Fujiwara et al., and Wu et al. that FAZ is not affected by age, gender, present and level of CKD, and present and level of diabetes (Fujiwara et al., 2017; Yeung et al., 2019; Wu et al., 2020; Oliverio et al., 2021). Therefore, FAZ may not be a sensitive marker to detect microvascular changes in patients with CKD.

On the other hand, VD and PD are significantly lower in patients with diabetic CKD when compared with the control. However, this difference is not observed when comparing the control with other causes of CKD (HPT group and AG group). The significant association between VD, PD, and diabetic CKD was still valid after controlling age as a covariate using Quade’s ANCOVA. However, we recognized the higher age distribution in the diabetic CKD compared to the control group, and this may pose some limitations in the ANCOVA analysis. The evidence of a strong correlation between HbA1c and FBS (significantly higher in the DM group) with VD and PD showed the present and higher level of diabetes would reduce the VD and PD in patients with CKD. The earlier studies on OCTA parameters in diabetes showed significant change in patients with DM and correlated with the level of DM control, FBS, and HbA1c (Ting et al., 2017). Urea and creatinine level (significantly higher in CKD groups), on the other hand, showed only a weak correlation with VD and PD. Based on our study, VD and PD are useful to differentiate the causes of CKD to diabetic vs. non-diabetic patients, but not useful to detect the presence of CKD in diabetic patients. This result was in line with the previous studies done by Dimitrova et al. (2017); Samara et al. (2017), and Yeung et al. (2019).

Macular volume (MV) was significantly different between control and CKD groups but no further difference between various causes of CKD. This is likely due to the difference in the age between control and CKD as macular volume is reported reduced by age by Eriksson and Alm (2009) and Song et al. (2010). The correlation between age and MV further supports a significant but weak association between age and MV. There is no strong correlation of MV with any of the CKD profiles studied here, namely urea, creatinine, UPCI, HbA1c, and FBS.

Overall, the significant difference in VD, PD, MV between the control group and the CKD group in this study was due to the combination of age difference and component of DM as one of the CKD subgroups. Further analysis in this study showed only VD and PD in the DM group were significantly lower after controlling age as a covariate. Looking at the previous studies with various results on the effect of different systemic vascular diseases on retinal vasculature, this study added further value by showing aging, DM, and its related parameters (HbA1c and FBS) pose more effect on the retinal vasculatures as measured by non-invasive OCTA.

The limitations of this study are not able to include some other systemic factors that may have implications on the retinal vasculature, which are blood pressure and body mass index (BMI). The effect of these two factors on retinal vasculature and OCTA parameters are so far inconclusive by previous studies. Yang et al. (2021) found no significant relationship between OCTA parameters with BMI and blood pressure. However, this finding was opposed by the results from Zhang et al. (2011) whereby the BMI correlated significantly with outer macular thickness. The other limitation of this study is the different distribution in the ethnicity of the study population because of the multi-ethnic society setting in Malaysia. The study population showed the unproportioned distribution of patients in the three groups of patients with CKD. The number of hypertensive CKD and autoimmune-related glomerulonephritis CKD were relatively low compared to the control group and diabetic CKD group as it was quite hard to recruit subjects with lupus nephritis and IgA nephropathy into this study as the nature of the disease prevalence.

## Conclusion

The fast and non-invasive nature of OCTA scanning provides a good opportunity to quantitatively measure the retinal microvasculature. Our study showed the meaningful reduction of VD and PD, particularly in patients with diabetes and CKD. However, the use of OCTA to screen or predict CKD in patients living with DM, hypertension, or autoimmune nephritis was not shown to be useful from this study.

## Data Availability Statement

The raw data supporting the conclusions of this article will be made available by the authors, without undue reservation.

## Ethics Statement

The studies involving human participants were reviewed and approved by UKM Medical Research and Ethics Committee under research code: GGPM-2019-023. The patients/participants provided their written informed consent to participate in this study.

## Author Contributions

WW, RMu, and RMo contributed to the conception and design of the study. MY contributed to the funding acquisition. MY, MO, KT, SH, and AM contributed to the data curation and formal analysis. MY and MO contributed to the writing-original draft. MY and WW contributed to the writing-review and editing. All authors contributed to the article and approved the submitted version.

## Conflict of Interest

The authors declare that the research was conducted in the absence of any commercial or financial relationships that could be construed as a potential conflict of interest.

## Publisher’s Note

All claims expressed in this article are solely those of the authors and do not necessarily represent those of their affiliated organizations, or those of the publisher, the editors and the reviewers. Any product that may be evaluated in this article, or claim that may be made by its manufacturer, is not guaranteed or endorsed by the publisher.
